# Evaluation of commercial rapid tests for fast and on-site detection of high-pathogenicity avian influenza H5 virus in poultry

**DOI:** 10.1128/spectrum.00654-25

**Published:** 2025-08-27

**Authors:** F. J. van der Wal, S. B. E. Pritz-Verschuren, E. A. Germeraad, A. de Bruin, S. M. de Boer, N. Beerens, J. L. Gonzales

**Affiliations:** 1 Wageningen Bioveterinary Research, Wageningen University & Research 4507 , Lelystad, the Netherlands; Erasmus MC, Rotterdam, the Netherlands

**Keywords:** avian influenza virus, HPAI, poultry, rapid test, on-site detection, diagnostic sensitivity, diagnostics

## Abstract

Since 2016, numerous outbreaks of high-pathogenicity avian influenza (HPAI) have occurred on poultry farms in Europe. Samples from suspected outbreaks are investigated using PCR at the national reference laboratories (NRLs) to detect the presence of the influenza virus. In the Netherlands, on occasion, multiple poultry farms needed to be investigated, sampled, and prioritized for testing at the NRL. For these and other situations, on-site pre-screening with rapid diagnostic tools could, in addition to visual inspections, provide additional information to support decisions on prioritization. This study focuses on lateral flow tests as rapid on-site diagnostic tools. Upon a review of available tests, five were selected and evaluated. Rapid tests from Anigen, Biopanda, and Clungene had the best limit of detection and could detect all hemagglutinin subtypes (H1–16). Testing respiratory and cloaca swabs from chickens and ducks experimentally infected with HPAI H5 virus revealed a relationship between the results of rapid tests and the sample’s viral load (Ct value of PCR). The Clungene rapid test had a diagnostic sensitivity (DSe) > 71% when Ct values were < 25, whereas the DSe was compromised when Ct values were > 28. A statistical model was built to describe this relationship and was applied to historic PCR data of samples from recent HPAI H5 outbreaks in the Netherlands. The predicted DSe of the Clungene rapid test was > 81% for respiratory and cloaca samples from ducks, turkeys, and chickens. These results indicate that rapid tests may serve as additional diagnostic tools for pre-screening samples suspected of avian influenza.

**IMPORTANCE:**

This study was performed to investigate whether rapid tests could serve as additional diagnostic tools for pre-screening samples suspected of avian influenza. Several rapid tests were acquired and tested for their analytical sensitivity. With a subset of these, swabs from chickens and ducks experimentally infected with avian influenza were investigated. A relationship of the test results with the viral load was modeled, and by using historic data from recent H5N1 outbreaks in The Netherlands, the predicted diagnostic sensitivity of a rapid test was > 81%. This suggests that rapid tests could have potential as supportive diagnostic tools in outbreak situations with avian influenza, but PCR by the national reference labs remains essential and obligatory for detection of avian influenza.

## INTRODUCTION

Avian influenza (AI), commonly known as bird flu, is an infectious disease in birds that is caused by the influenza virus type A. Based on the surface proteins hemagglutinin and neuraminidase, different avian influenza virus (AIV) subtypes can be distinguished, which are defined by combinations of hemagglutinin (H) and neuraminidase (N); 16 H (H1–H16) and 9 N (N1–N9) subtypes are circulating in birds. AIVs are classified as low pathogenicity (LPAIV) or high pathogenicity avian influenza viruses (HPAIV) (1). The latter contain H5 or H7 hemagglutinins with a cleavage site susceptible to furin-like proteases and either emerge from LPAIV upon infection in poultry (2, 3) (circulating only in poultry) or, in case of most recent H5 strains, are circulating in both wild birds and poultry (4). Since 2014, the HPAI H5Nx virus of the clade 2.3.4.4 (5) has been spreading worldwide. H5N1 virus of clade 2.3.4.4.b (6) caused a large panzootic of HPAI, starting in 2020 with devastating consequences for poultry and wild birds and with an increasing frequency of spillover events to mammals (7–13).

In the European Union, HPAIV in poultry is subject to control according to the Animal Health Law: for suspected cases, diagnostics are carried out, as prescribed by the WOAH, with PCR and are confirmed and subtyped with other molecular diagnostics (14). Upon a suspicion of infection, 20 dead or sick birds per poultry house are sampled by taking both throat and cloaca swabs. Upon arrival at the National Reference Laboratory (NRL), these swabs are tested in pools of five for the presence of viral RNA, using RT-PCR targeting the gene encoding the matrix protein of AIV. Positive samples are further investigated for the presence of H5 or H7 subtypes and the sequence of the hemagglutinin cleavage site; a polybasic cleavage site is characteristic of HPAIV. If samples are found to contain HPAIV, all birds on the positive farm are culled. Rapid diagnosis and subsequent implementation of measures are essential to prevent spreading between poultry farms, particularly in the poultry-dense areas of the Netherlands (15).

In 2022, a human influenza rapid test was used for testing birds by a Dutch animal rescue organization (Dierenambulance De Ronde Venen/Amstelland) (16). Results were not published, but they provoked interest in investigating the performance characteristics of rapid tests for influenza in poultry and their application as diagnostic tools for pre-screening purposes during emergencies. If rapid tests are sufficiently sensitive and specific for AIV, the use of rapid tests for on-site pre-screening of poultry may be useful when multiple (suspected) outbreaks on poultry farms occur simultaneously, especially in areas densely populated with poultry farms where the risk of epidemics (sustained farm-to-farm transmission) is high (15). Pre-screening with rapid tests may help to prioritize available manpower and resources required for sampling and testing by PCR.

Rapid on-site tests that detect influenza A viruses are available in the same format as the well-known rapid tests for coronaviruses. These tests are based on direct detection of viral antigens by antibodies in a lateral flow immunoassay (LFIA) and can be used on-site, outside of a laboratory. However, LFIAs are less sensitive than PCR (17) as the tests can only detect molecules that are present (i.e., viral antigens), whereas during a PCR test, the target molecule (i.e., genetic material of the virus) is multiplied, thereby boosting the sensitivity of the test. Nevertheless, the use of rapid tests in the format of LFIAs during the COVID-19 pandemic has shown that even diagnostic tools with suboptimal sensitivity can be useful (17). Over the years, various rapid tests for influenza virus have been used in studies to detect AIV in birds (e.g., references 18–20). The rapid tests are for either human samples or for swabs taken from poultry and wild birds and are, in general, not capable of subtyping influenza A viruses, as most of these rapid tests target the nucleoprotein.

Currently, a number of these rapid tests for the detection of influenza A virus are on the market, but results are difficult to compare as the performance characteristics of the rapid tests are not established according to identical procedures. The objectives of this study were (i) to have an overview of available rapid tests that may be suitable for detecting AIV in poultry, (ii) to assess under controlled conditions the diagnostic performance of user-friendly rapid tests for detection of HPAI H5 virus using samples from infected poultry, (iii) to investigate whether these rapid tests detect all 16 H subtypes of AIV, and (iv) to predict the diagnostic sensitivity (DSe) of a rapid test in the field, based on PCR results obtained during the recent HPAI H5N1 epidemics.

## MATERIALS AND METHODS

### Selection of rapid tests

A review of rapid tests for influenza was performed. Overviews were prepared of rapid tests for detecting influenza A virus in poultry, in human samples, and published use of rapid tests for detecting influenza in poultry. Finally, a preselection of rapid tests was made—for further laboratory evaluation—based on the current availability and presumed ease of use.

### Study design and samples for the evaluation of rapid tests’ diagnostic performance

Laboratory evaluation of diagnostic performance followed a sequential approach. First, five rapid tests were assessed for their analytical sensitivity. Second, three tests with the highest analytical sensitivity were selected to further assess their inclusivity and diagnostic performance under laboratory conditions. In addition, we assessed the relationship between the diagnostic sensitivity of one rapid test and the virus load in the sample (measured by real-time PCR). This relationship was used to predict the potential diagnostic sensitivity of the rapid test for use in the field.

The analytical sensitivity of rapid tests was investigated using a stock of an HPAI H5N1 virus isolate (A/chicken/Netherlands/21038165-006010/2021) from an outbreak in 2021 in a chicken farm. Inclusivity of rapid tests for H subtypes was investigated with virus stocks from 18 different AIVs, that is, 16 LPAIVs with one virus per H subtype (H1-H16) and two HPAIVs (H5 and H7) (Table 1, left panel).

**TABLE 1:** Viruses used and inclusivity of rapid tests^*a*^

Virus	Name	Ct	Clungene		Biopanda		Anigen	
H1N5	A/turkey/Netherlands/07014290/2007	19.4	+	+	+	+	+	+
H2N7	A/chicken/Netherlands/14003005/2014	20.1	+	+	+	+	+	+
H3N8	A/mallard/Netherlands/11015220/2011	20.8	+	+	+	+	+	+
H4N6	A/mallard/Netherlands/09014545/2009	20.5	+	+	+	+	+	+
H5N1 HP	A/chicken/Netherlands/21038165-006010/2021	18.3	+	+	+	+	+	+
H5N2 LP	A/chicken/Netherlands/17014215-026030/2017	20.3	+	+	+	+	+	+
H6N8	A/duck/Netherlands/14016168/2014	21.6	+	+	+	+	+	+
H7N7 HP	A/chicken/Netherlands/1/2003	21.1	+	+	+	+	+	+
H7N9 LP	A/chicken/Netherlands/16007311-058062/2016	20.0	+	+	+	+	+	+
H8N4	A/chicken/Netherlands/11004004/2011	19.0	+	+	+	+	+	+
H9N2	A/chicken/Netherlands/10020245/2010	23.5	+	+	+	+	+	+
H10N3	A/mallard/Netherlands/22011112-006/2022	20.0	+	+	+	+	+	+
H11N6	A/duck/England/1/56	19.2	+	+	+	+	+	+
H12N5	A/mallard/Netherlands/15011910/2015	18.2	+	+	+	+	+	+
H13N3	A/black-headed gull/Netherlands/72/2008	19.9	+	+	+	+	+	+
H14N7	A/herring gull/Netherlands/08007097/2008	22.9	+	+	+	+	+	+
H15N9	A/shearwater/West Australia/2576/79	18.4	+	+	+	+	+	+
H16N3	A/black-headed gull/Netherlands/08016893/2008	22.7	+	+	+	+	+	+
	NC 1	−	−	−	−	−	−	−
	NC 2	nd	−	nd	−	nd	−	nd

^*a*^ Ct values given are the average (max. SD 0.2) of two runs of the M-PCR of virus stocks diluted 1:100 in 2.95% tryptose phosphate buffer (TFB)**.** The same samples were tested twice with three rapid tests. The H5N1 HP isolate was also used for investigating analytical sensitivity. Symbols and abbreviations: +, positive; −, negative; HP, high pathogenicity; LP, low pathogenicity; NC 1, negative control 1 (TFB); NC 2, negative control 2 (negative allantois liquid 1:100 in TFB); nd, not determined.

For the assessment of the diagnostic performance, a prospective (opportunistic) study approach was taken where true-positive and -negative samples from chickens and broiler ducks were obtained from an infection experiment performed for another study (21), where birds were challenged with either of two recent HPAI H5N1 viruses isolated from poultry in the Netherlands during the periods 2020–2021 and 2021–2022.

To assess diagnostic specificity (DSp) of rapid tests, true-negative samples were taken from 28 specific pathogen-free (SPF) chickens and 28 conventional Pekin ducks confirmed negative for AIV by PCR. The choana was swabbed instead of the trachea as this is less intrusive for live birds. Next to the choana swabs, cloaca swabs were taken. For chickens, the swabs for investigating rapid tests were the first swabs taken from choana and cloaca; for ducks, this was the second set of swabs taken from choana and cloaca (i.e., after swabs for PCR).

To assess the DSe of rapid tests, true-positive swab samples were taken, during necropsy, from 16 chickens at 2 days post-infection (dpi) and eight Pekin ducks at 3 dpi. Before necropsy (2–8 hours), swabs from these birds (while still alive) were taken from the choana and cloaca for PCR confirmation of their infection status. During the necropsy, samples were taken from the trachea (not choana) and cloaca for the rapid tests. Swabs for the most analytically sensitive rapid test were taken first, and then alternating swabs for the other two tests were taken. For each rapid test, swabs supplied with the corresponding rapid test were used.

For the prediction of the expected DSe of a rapid test for field samples, retrospective data (from October 2021 to January 2023) were used. The data comprise Ct values of PCR-positive pooled samples from 88 farms confirmed to be infected with HPAI H5 virus. Samples from these farms were submitted to the NRL for confirmation of outbreak suspicions (*n* = 712 pools from 85 farms), for screening during outbreak response (e.g., contact tracing, *n* = 44 pools from six farms), or for differential diagnosis (*n* = 4 pools from two farms). The latter are samples submitted for diagnosis of diseases other than HPAI. In total, retrospective data were available for 400 pools of trachea swabs and 360 pools of cloaca swabs, where each pool consisted of 5 swabs. In addition to the type of swab, information on the poultry species was available. Given the opportunistic nature of this study, no preliminary estimation of sample sizes was made.

### Diagnostic methods

#### Rapid tests for influenza virus

Following a review and preliminary assessment of available rapid tests in the market, five tests were selected for this study. The investigated rapid tests were from Abbexa (Avian Influenza Virus Antigen Rapid Test Kit, abx092015), Anigen (AIV Ag Test Kit, RG1501MH), Biopanda (Avian Influenza Virus [AIV] Antigen Rapid Test, RAPG-AIV-001), Clungene (COVID-19/Influenza A + B Antigen Combo Rapid Test, IrIDq325-B025), and Quidel (QuickVue Influenza A + B Test, 20182IN). The tests were performed according to the manufacturer’s protocol, except when investigating the analytical sensitivity, for which no swabs were used, and virus dilutions were added directly to the assay buffer. For each rapid test, the maximum number of prescribed drops was added, except for Quidel test strips, which were placed directly into the prepared sample. To assess analytical sensitivity, results were promptly assessed visually by *one* technician after the prescribed incubation periods. For assessing diagnostic sensitivity and specificity, birds were sampled with swabs provided with the rapid tests, transported in tubes to the laboratory, and tested according to the test’s manuals. Here, the diagnostic procedures and reading of the rapid tests (results) were performed by *two* technicians who were aware of the expected infection status of the birds.

#### PCR (reference method)

Samples used in this study were confirmed by a quantitative real-time RT-PCR that targets the gene encoding the matrix protein of AIV, as described (22).

### Analyses

#### Analytical sensitivity

The analytical sensitivity of five rapid tests was assessed using a dilution series of a virus suspension to quantify the detection limits. The rapid tests were examined with a suspension of HPAI H5N1 virus isolate (see Table 1), which had a concentration of 10^9,94^ EID_50_/mL. Dilution series with a semi-logarithmic dilution (0.5 log_10_ steps) were made in 2.95% tryptose phosphate broth (TFB). For each rapid test, an arbitrary quantity of 20 µL was added to the supplied aliquots of assay buffer. This was done to test the same quantity of virus in each test by circumventing the effects of the different swabs supplied with the tests. Differences in analytical sensitivity were established by testing dilution series (and a negative control) in quadruplicate. The limit of detection (LOD) of each test (the point at which 50% of the tests are expected to be positive) was calculated using the Spearman-Kärber formula (23).

#### Inclusivity of H subtypes by testing diluted virus stocks

For three rapid tests that were assessed to have the highest analytical sensitivities (see Results), virus stocks in allantois liquid of 16 different H subtypes and both HPAI and LPAI H5 and H7 viruses were used to investigate inclusivity of H subtypes. These rapid tests target the nucleoprotein of influenza viruses. Stocks of the 18 viruses (Table 1) were diluted 1:100 in 2.95% tryptose phosphate buffer (TFB). TFB and negative allantois liquid (1:100 in TFB) were used as negative controls. Rapid tests were performed as described above. The 1:100 dilutions were also tested in PCR to get an indication of the amount of genetic material in the samples tested. Rapid tests and PCRs were performed in duplicate.

#### Diagnostic specificity and diagnostic sensitivity

Based on the assessment of the analytical sensitivity, the majority of true-negative birds (20 out of 28 chickens and 20 out of 28 ducks) were sampled for testing with the most sensitive rapid test; for each of the two other selected rapid tests, four birds of each species were sampled and tested (Table 2). Samples from all 16 true-positive chickens and all eight infected ducks were sampled for testing with the most sensitive rapid test. After that, birds were resampled for testing with the two other selected rapid tests: with each of the two other selected rapid tests, half of the birds of each species were tested (Table 2).

**TABLE 2:** Sampling and testing of birds^*a*^

	AI-negative birds			AI-infected birds		
Species	Swabs	Test	Birds (N)	Swabs	Test	Birds (N)
Chicken	–^*b*^	–	–	1st set: choana & cloaca	PCR	16 (all)
	1st set: choana & cloaca	Clungene	20	2nd set: trachea & cloaca	Clungene	16 (all)
		Biopanda	4	3rd set: trachea & cloaca	Biopanda	8
		Anigen	4		Anigen	8
Duck	1st set: choana & cloaca	PCR	28 (all)	1st set: choana & cloaca	PCR	8 (all)
	2nd set: choana & cloaca	Clungene	20	2nd set: trachea & cloaca	Clungene	8 (all)
		Biopanda	4	3rd set: trachea & cloaca	Biopanda	4
		Anigen	4		Anigen	4

^*a*^ Overview of sampling of birds and the diagnostic methods used. From each bird, for each test, one swab was taken per location.

^*b*^ “–”, not applicable.

The DSp and DSe were estimated as the proportion of positive or negative rapid test’s classifications of truly positive (infected birds following experimental infection and confirmed by PCR) or truly negative birds (uninfected). The corresponding 95% confidence intervals were calculated using the Clopper-Pearson method (exact confidence intervals) (24), which is suitable when dealing with small numbers of samples. Results were omitted when rapid tests were not executed correctly (see the legend to Table 3 for details).

**TABLE 3:** DSp of rapid tests with samples from AI-negative chickens and Pekin ducks^*a*^

					Results					
Species	AI-Neg. status	Swab^*b*^	Test	N	Rej.	Pos.	Neg.	Neg./total	DSp (%)	95% CI (%)
Chicken	SPF	Choana	Clungene	20	0	0	20	20/20	100.0	83.9–100.0
			Biopanda	4	0	0	4	4/4	100.0	51.0–100.0
			Anigen	4	0	0	4	4/4	100.0	51.0–100.0
		Cloaca	Clungene	20	2^*c*^	1^*e*^	17	17/18	94.0	74.2–99.0
			Biopanda	4	0	0	4	4/4	100.0	51.0–100.0
			Anigen	4	0	0	4	4/4	100.0	51.0–100.0
Duck	PCR negative	Choana	Clungene	20	1^*d*^	0	19	19/19	100.0	83.2–100.0
			Biopanda	4	0	0	4	4/4	100.0	51.0–100.0
			Anigen	4	0	0	4	4/4	100.0	51.0–100.0
		Cloaca	Clungene	20	0	0	20	20/20	100.0	83.9–100.0
			Biopanda	4	0	0	4	4/4	100.0	51.0–100.0
			Anigen	4	0	0	4	4/4	100.0	51.0–100.0

^*a*^ Choana and cloaca swabs from AI-negative birds were investigated with the three rapid tests. For the most sensitive test (see Table S3), more swabs were investigated. The results are presented together with the corresponding diagnostic specificity and confidence interval (95%). N, number; Rej, rejected; Pos, positive; Neg, negative; CI, confidence interval.

^*b*^ For respiratory swabs from live birds, choana swabs were taken.

^*c*^ Incorrect volume added to test due to blocking of droplet pipet and/or uncontrolled release of sample material upon applying increased pressure to force release of droplets.

^*d*^ Swab was accidentally removed from the assay buffer before the required incubation period ended.

^*e*^ Sample was found positive for both influenza A and B, retesting resulted in the same outcome and the transport container of this swab was tested negative by PCR.

#### Relationship between diagnostic sensitivity and Ct value

The relationship between a sample’s PCR Ct value and the probability of detection (diagnostic sensitivity) in the rapid test was further explored. To this end, a graphical exploration of this relationship was performed, after which this relationship was quantified by fitting a logistic regression model. In this model, the rapid test result (positive or negative) of each sample is the response variable, and the sample’s Ct value is the explanatory variable. Given the limited number of samples, variables such as poultry species or type of swab were not taken into account. Hence, in this model, it is assumed that the sensitivity of the rapid tests is only influenced by the virus load (Ct value) in the sample.

#### Predicting the effectiveness of rapid tests if applied to field samples

The developed logistic regression model was used to predict the probability of detecting HPAIV (sensitivity of the test) in positive field samples by using their Ct values. Subsequently, the predicted probabilities were averaged for each combination of bird species and swab type. These average probabilities represent the predicted diagnostic sensitivity of the rapid test. See Yellowlees and Perry (25) for a detailed description of this approach.

## RESULTS

### Selection of rapid tests

A review of rapid tests for influenza resulted in three comprehensive overviews with, respectively, rapid tests for influenza A virus in poultry, influenza in human samples, and tests with published use for detection of influenza in poultry (Table S1A through C). A preselection of nine tests was made based on current availability and in part on published (and unpublished) use in poultry or birds. The preselection (Table S1D) consisted of two relatively unknown tests for poultry (Abbexa and Biopanda) and two well-established tests for poultry (Megacor and Novidia), all available from manufacturers in Europe or the United States, and a fifth test for poultry from an Asian manufacturer (Anigen), which has been used in research for over a decade (see Table S1C). In addition, the preselection contains four rapid tests for human samples, three of which have also been described in the literature for use in poultry (Abbott, Remel, Quidel; see Table S1C), and one that has been used for Dutch wild birds (see Introduction). Prior to testing, and solely based on availability and presumed ease of use, five rapid tests were selected for this study. Decisions to accept or reject tests are indicated in Table S2. These are the rapid tests that are expected to be user-friendly and suitable for on-site use during outbreaks, that is, the rapid tests from Abbexa, Biopanda, Anigen, Quidel, and Clungene.

### Analytical sensitivity of five rapid tests

For the five selected rapid tests, the analytical sensitivity was established. This was done by using a dilution series of virus suspensions to determine the LOD. Calculated LODs ranged from 10^8.8^ EID_50_/mL to 10^6.3^ (Table S3). In this experimental setup, the Clungene test was the most sensitive rapid test, with the estimated LOD being 10^6.3^ EID_50_/mL, followed by those of Biopanda (10^6.7^ EID_50_/mL) and Anigen (10^6.7^ EID_50_/mL). The tests from Quidel (10^7.2^ EID_50_/mL) and Abbexa (10^8.8^ EID_50_/mL) showed the lowest analytical sensitivity and were excluded from further assessments.

### Inclusivity of three rapid tests for H subtypes

The rapid tests from Anigen, Biopanda, and Clungene were investigated for their capability to detect a broad panel of viruses with different H subtypes. The average Ct values of the used virus dilutions ranged from 18.2 to 23.5. The three rapid tests successfully detected all 18 viruses included in the test panel (Table 1).

### Diagnostic specificity

Swabs from uninfected chickens and ducks were tested with three rapid tests, with focus on the most analytically sensitive rapid test, that is, the test from Clungene (see Table 2). Of all performed tests, three were rejected because they were not executed correctly (incorrect volume added to the test or incorrect incubation time of the swab (Table 3). The DSp (95% CI) of the Clungene rapid test in chickens was 100.0% (CI 83.9%–100.0%) for choana swabs and 94.0% (74.2%–99.0%) for cloaca swabs. In Pekin ducks, the DSp was 100.0% for both the choana swabs (CI 83.2%–100.0%) and the cloaca swabs (CI 83.9%–100.0%). The lower DSp with chicken cloaca swabs was because of one false-positive sample in this set of swabs; oddly, this sample also showed a signal on the test line for influenza B virus. The rapid tests from Anigen and Biopanda correctly classified all samples from the four chickens and ducks sampled for these tests as negative. Given the very small sample size for these tests (only four swabs per category), their DSp values have broad confidence intervals (Table 3).

### Diagnostic sensitivity

Trachea and cloaca swabs from chickens and ducks experimentally infected with AIV were used to assess the DSe of rapid tests. Swabbing and testing were as indicated in Table 2. For chicken samples, the DSe (95% CI) for the Clungene rapid test was 93.3% (CI 70.2%–98.8%) for trachea samples and 92.9% (68.5%–98.7%) for cloaca samples (Table 4). For the Biopanda rapid test, the DSe for trachea and cloaca swabs was 100.0% (CI 64.6%–100.0%) and 71.4% (CI 35.9%–91.8%), respectively. For Anigen, the DSe was 87.5% (CI 52.9%–97.8%) for both types of swabs. In ducks, the DSe of all three tests was 100.0% with confidence intervals ranging from 67.6% (Clungene) or 51.0% (Anigen, Biopanda) to 100.0% for trachea swabs, but 50.0% (CI 21.5%–78.5%) (Clungene) or 0.0% (CI 0.0%–49.0%) (Anigen, Biopanda) for cloaca swabs.

**TABLE 4:** DSe of rapid tests with samples of PCR-positive chickens and ducks^*a*^

				Results					
Species	Swab^*b*^	Test	N^*c*^	Pos.	Neg.	Pos./total	Mean Ct (range)	DSe (%)	95% CI (%)
Chicken	Trachea	Clungene	15	14	1	14/15	23.9 (19.0–36.5)	93.3	70.2–98.8
		Biopanda	7	7	0	7/7	24.6 (19.0–36.5)	100.0	64.6–100.0
		Anigen	8	7	1	7/8	23.3 (19.9–26.0)	87.5	52.9–97.8
	Cloaca	Clungene	14	13	1	13/14	24.5 (21.4–28.9)	92.9	68.5–98.7
		Biopanda	7	5	2	5/7	24.8 (22.2–28.9)	71.4	35.9–91.8
		Anigen	8	7	1	7/8	24.3 (21.4–28.0)	87.5	52.9–97.8
Duck	Trachea	Clungene	8	8	0	8/8	20.9 (19.3–24.8)	100.0	67.6–100.0
		Biopanda	4	4	0	4/4	22.1 (19.8–24.8)	100.0	51.0–100.0
		Anigen	4	4	0	4/4	19.8 (19.2–20.7)	100.0	51.0–100.0
	Cloaca	Clungene	8	4	4	4/8	28.5 (26.6–29.8)	50.0	21.5–78.5
		Biopanda	4	0	4	0/4	28.2 (26.6–29.3)	0.0	0.0–49.0
		Anigen	4	0	4	0/4	28.8 (27.8–29.8)	0.0	0.0–49.0

^*a*^ Trachea and cloaca swabs from experimentally infected birds were investigated with three rapid tests. For the most sensitive test, more samples were investigated. Results given are the positive test results of three rapid tests over the number of tests performed, corrected for rejected birds (not positive by PCR) and rejected rapid test results (not executed correctly, see Table 3). In addition, the average Ct and range of Ct values of the corresponding PCRs are given. For the three rapid tests, the calculated DSe and 95% CI are given for the various categories. CI, confidence interval.

^*b*^ Trachea swabs were taken from positive (deceased) birds.

^*c*^ The number of PCR-positive birds that were tested.

**Fig 1:**
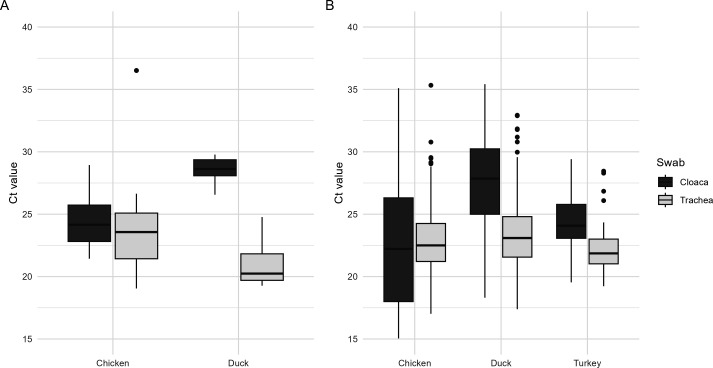
Distribution of Ct values of trachea and cloaca swabs. Ct values are summarized for trachea and cloaca swabs from chickens and ducks experimentally infected with HPAI H5N1 (A) and from PCR-positive Dutch field samples (chicken, duck, and turkey) submitted for testing from October 2021 to January 2023 (B).

The Ct values’ distribution of these samples is shown in Fig. 1A. Trachea swabs from both chickens and ducks had average Ct values < 25 and were nearly all positive in the rapid tests (44 out of 46 for all tests together; Table 4). Similarly, cloaca swabs from chickens had Ct values < 25 and were nearly all positive in the rapid tests (25 out of 29). This was not the case for cloaca swabs from ducks, which had higher average Ct values (>28) and where only 4 out of 12 tested samples were positive in the rapid tests. Taking all these results together, they suggest a dependency of the DSe of the rapid tests on the sample’s viral load.

### Quantifying the DSe as a function of the Ct value

As mentioned above, an apparent dependency of the DSe of the rapid tests on the sample’s viral load (Ct value) was observed. This suggests that it may be possible to quantify the DSe obtained from a rapid test as a function of Ct values obtained from a PCR test. We selected the data from the Clungene rapid test, which had the highest number of samples tested, to fit a logistic regression model, where the DSe is predicted using information on the sample Ct value as follows:


(1) $$Diagnostic\;sensitivity=\frac{1}{1+e^{-\left(5.661-0.149*Ct\right)}}$$

**Fig 2:**
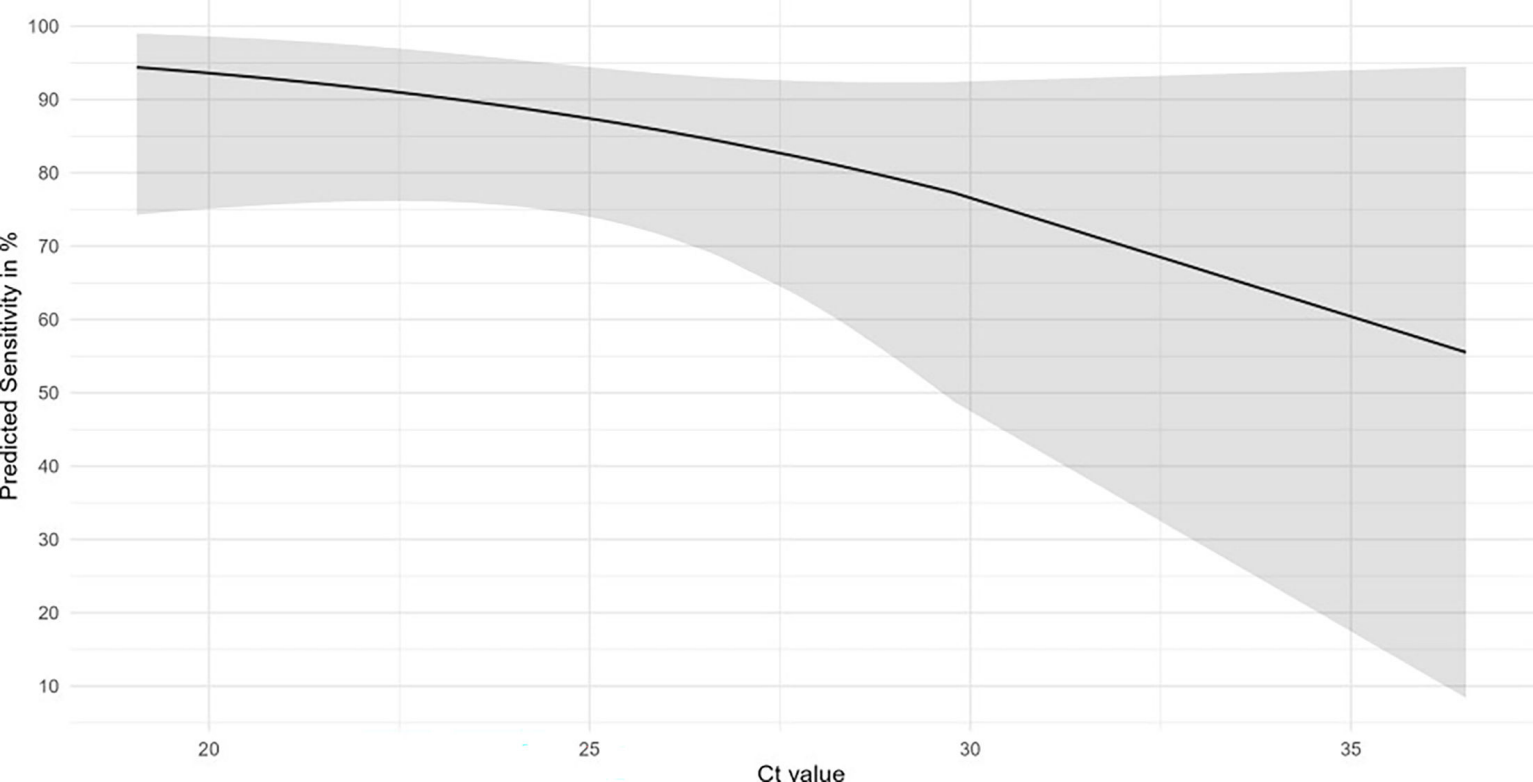
Modeled DSe of the Clungene rapid test. The predicted average DSe (black line) and its 95% confidence interval (gray area) are given for the Clungene rapid test. The diagnostic sensitivity is modeled using Ct values of the trachea and cloaca swabs from the H5N1 experimentally infected birds.

This model predicts that testing samples with Ct ≤ 25 will result in a DSe (95%CI) of 87.4% (CI 74.0%–94.4%) or higher (Fig. 2). Above Ct 25, the estimated DSe decreases, and the CI becomes increasingly broader. At Ct 28, the predicted mean diagnostic Se is 81.6% (CI 61.2%–92.4%). With Ct > 28, the reliability becomes low, as can be inferred from the broad confidence intervals, which are due to the limited number of PCR-positive samples with Ct values over 28 (see Fig. 1A).

### Predicting diagnostic performance of the Clungene rapid test for field samples

Available historic data for field samples consisted of Ct values for trachea and cloaca pools from chickens, ducks, and turkeys. The distribution of Ct values of these pools is shown in Fig. 1B. Similar to the experimental infection (Fig. 1A), the Ct values of cloaca pools from ducks are in general higher than Ct values from trachea pools from all species, and higher than cloaca pools from chickens and turkeys (Fig. 1B). The Ct values from these pools were used to inform the developed model (equation 1) to predict the DSe of the Clungene rapid test if it were applied to field samples. Table 5 summarizes the mean Ct values per bird species and swab type as well as the predicted DSe of the Clungene rapid test for these pools. For pools of trachea swabs, the predicted DSe (95% CI) was 90.1% (CI 74.1%–96.5%) for chicken, 88.5% (CI 71.1%–96.0%) for ducks, and 90.5% (CI 74.0%–96.8%) for turkeys. Looking at the lower 95% confidence limit of the predicted DSe, one could expect that the DSe of the Clungene rapid test, when applied to trachea samples in the field, would at least be ≥ 70% for all bird species. For chickens and turkeys, the predicted DSe values of cloaca samples were similar to that of trachea samples; however, for ducks, the DSe dropped to 81% (56.7%–94.1%), reflecting the higher Ct values observed in cloaca samples of ducks (see Fig. 1B). The Ct value distribution and predicted DSe when considering the source of the samples (reason for testing, species, swab type) are shown in Table S4 and Fig. S1.

**TABLE 5:** Predicted DSe of the Clungene rapid test for PCR positive Dutch field samples^*a*^

Species	Swabs pooled	Number of pools	Mean Ct (range)	DSe (%)	95% CI (%)
Chicken	Trachea	275	22.8 (17.0–35.3)	90.1	74.1–96.5
	Cloaca	269	22.6 (15.1–35.1)	88.9	68.3–96.5
Duck	Trachea	98	23.7 (17.4–32.9)	88.5	71.1–96.0
	Cloaca	71	27.3 (18.3–35.4)	81.1	56.7–94.1
Turkey	Trachea	27	22.5 (19.2–28.4)	90.5	74.0–96.8
	Cloaca	20	24.3 (19.5–29.4)	87.9	71.7–95.3

^*a*^ The modeled diagnostic sensitivity of the Clungene rapid test (Fig. 2) was used to predict results of the Clungene rapid test if it had been used on PCR-positive field samples. PCR data used are of field samples that were tested in the period October 2021–January 2023. Predicted DSe and CI are given per bird species and per type of swab. In addition, the mean Ct values (and the range) of the corresponding historic PCR data are given.

## DISCUSSION

This study aims to investigate whether rapid tests can play a role as screening tests for the absence or presence of AIV, particularly HPAIV, in poultry. By no means, the intention is to advocate the replacement of PCR by rapid tests. Following a review of available rapid tests, five rapid tests were selected for further study. In a laboratory setting, the rapid tests from Clungene, Anigen, and Biopanda have the best LOD. Within a set of AIV with different H subtypes, these three rapid tests all detect AIV with H subtypes H1-16, including HPAI H5 and H7 variants. A relationship between the outcome of the rapid tests and the viral load (measured as Ct values) present in samples was observed, with rapid tests showing high diagnostic sensitivity (DSe) when the Ct values of the samples were ≤ 25. The latter was the case for most of the choana swab samples taken from experimentally infected chickens and ducks. For Ct values > 28, which was the case for most of the cloaca swab samples taken from ducks, the DSe was low. Based on the relationship between the sample’s Ct value and the probability of detection, information on the Ct value of samples collected from outbreak investigations in the field was used to predict the DSe of the Clungene rapid test. The average Ct values of the field samples (trachea and cloaca swabs) were< 25, and the predicted DSe of this rapid test was > 81% for all types of samples, indicating that rapid tests could be fit for use in outbreak investigations as pre-screening tests. Given the imperfect DSe of rapid tests, their application is appropriate only at the flock level (26). To mitigate the risk of false-negative results, it is essential to sample and test multiple sick or dead birds from the same flock, thereby increasing the likelihood of detecting at least one positive bird if infection were present.

The inventory of rapid tests for influenza A virus led to a selection of tests that are both available on the market and user-friendly. The LODs of rapid tests are often provided by the manufacturer, but results are difficult to compare as the LODs are not established with the same virus strains or are expressed in different units (see Table S2). Therefore, in this study, the analytical sensitivities of five selected rapid tests were investigated with dilution series of one H5N1 clade 2.3.4.4.b virus. A factor that will affect the analytical sensitivity of a test is how well the supplied swabs absorb analytes and release them again in the assay buffer (27). In the present study, this was circumvented by using a fixed volume of virus dilution for each test. This allowed the assessment of tests, where “tests” is defined as combinations of a lateral flow device and its corresponding protocol, for there are differences in the volumes of the assay buffer provided and the number of drops that are to be added to the device. This is relevant for the users of the rapid tests in the field, as it would be pragmatic to use the swabs that are in use for PCR testing also for the rapid tests. The swabs used may, however, affect assay performance and may therefore not result in the same analytical sensitivity as established in this research. It is worth noting that for this study, individual birds were tested for the PCR and the rapid test, while field samples taken for PCR are pooled (*n* = 5). Further research to address this matter is required. In addition, while performing the tests, it was noted that interpretation of test results is difficult when the dilution series reaches the limit of detection. The highest dilutions can result in very weak signals that, in the laboratory, under good lighting conditions, are just perceivable by the naked eye but do not appear on photographs. It is therefore likely that sensitivities established in a laboratory study are difficult to reproduce in the field, where tests are performed under less optimal conditions.

The tests from Anigen, Biopanda, and Clungene recognized isolates of all H subtypes. Although the tests are based on the detection of nucleoprotein, the rationale for composing a panel with different H subtypes was to create a broad panel of influenza viruses that have in common that they are isolated from birds. The observed broad inclusivity of the tests is expected as they all target the nucleoprotein, which is a highly conserved protein (28) that is widely used for detecting AIV or antibodies against AIV (14). This broad inclusivity of the rapid tests is necessary as in the past, outbreaks have occurred with other subtypes (22, 29, 30) than HPAIV H5N1, which potentially could happen again.

A high DSp for all three rapid tests was observed. The analysis with the Clungene rapid test indicated that this DSp might be lower for cloaca swabs than for choana swabs. A cloaca sample that was false positive for influenza A virus was also positive for influenza B virus in the Clungene rapid test. Maybe this is indicative of a component in this particular cloaca sample that mediates aspecific binding of the conjugated gold nanoparticles of the test to immobilized antibodies in the Clungene test. Notably, the two other tests (that do not have a test line for influenza B) are on the market for use with avian samples and may be better suited to avoid aspecific interactions in cloaca samples, whereas the Clungene test is designed for human samples, not for cloaca swabs from poultry. As for the Biopanda and Anigen rapid tests, the limited sample size evidently limits the ability to draw conclusions on the DSp, and even though no false positives were detected, the possibility that cloaca samples could affect the DSp of these other rapid tests cannot be discarded. Further testing on field samples will allow quantification of the actual DSp of rapid tests when applied to trachea and cloaca swabs from poultry.

Exploring the distribution of Ct values of the experimental samples suggested that the DSe of the rapid tests depends on the samples’ Ct value rather than the bird species (chicken or ducks) or swab type (choana or cloaca). Furthermore, we also explored the Ct values distribution of field samples (tested as pools of five swabs) collected from confirmed HPAIV outbreaks in poultry farms in the Netherlands. The Ct values distribution of the experimental samples used for the assessment of the DSe was similar to the Ct values distribution observed in samples from the field. The average Ct values in respiratory samples were <25 in both experimental (choana) and field (trachea) samples (Tables 4 and 5), and the average Ct values in duck cloaca samples >26 in both experimental and field samples. Therefore, it is likely that the DSe estimated with experimentally infected birds will reflect the DSe of the test if it were to be applied in the field during an outbreak. If a sensitive test (such as the Clungene rapid tests with an expected DSe of circa 90%) is used in the field, then the most suited target populations for sampling would be dead or sick animals, which are likely to shed high levels of virus (reflected by low Ct values in PCR). Sampling dead animals at a determined frequency is a surveillance strategy suggested for early detection of infected poultry flocks so that these flocks can be removed before they spread infection to other flocks during epidemics (31). Application of a rapid test in the field may contribute to early detection and allow for prioritization in outbreak situations preceding PCR diagnostics at the NRL.

In addition to training of personnel, there are several practical aspects that need attention before rapid tests can be applied in the field during outbreaks. First, it must be determined how many animals need to be tested for a sound assessment. Currently, swabs for PCR are pooled before testing (per suspected outbreak, 20 trachea and 20 cloaca swabs, 5 swabs per pool), but individual swabs are used for the rapid test. Second, swabs that are included with the rapid tests are larger than the swabs used for the present routine sampling, which are relatively small and may better match the size of the various cavities in poultry that are sampled. Although swabs can hugely differ in efficiency of picking up material (27), a practical approach would be to use the swabs now used for sampling in the current system. A third consideration concerns testing of trachea and/or cloaca swabs. Despite the expected lower DSe in cloaca swabs, it is wise to test both trachea and cloaca swabs in the field. The reason is that respiratory shedding during the first days of infection can shift to shedding in the cloaca later in infection (32), which will affect the amount of virus that is present in the different sample types. Therefore, preferably both trachea swabs and cloaca swabs are tested, as advised for PCR (14). A final point that needs consideration is that the read-out of the tests around the LOD may be difficult when signals are weak. This may be circumvented by testing multiple animals and by sampling obviously sick birds that are likely to shed the virus at high levels. Nevertheless, it is conceivable that the DSe of rapid tests under field conditions is lower, especially in an environment that is suboptimal for performing tests. So, if rapid tests are going to be applied, conditions on site should be such that the tests can be carried out according to protocol, and that there is sufficient light to assess test results. Concerning the latter point, the use of relatively cheap (and therefore disposable) equipment to read tests could be considered.

The potential for batch-to-batch variation in the sensitivity of rapid tests should also be considered. Although we did not assess batch-to-batch variation in the current study—tests from one batch were used—the analytical sensitivity of a newly acquired batch of Clungene rapid tests for an ongoing study in wild birds was assessed. Using the same procedures as described in this study, the analytical sensitivity was found to be identical (data not shown). Nevertheless, if rapid tests are to be routinely used for surveillance, assessing the analytical sensitivity of each new batch before the tests are deployed in the field is advisable.

This study is limited by the fact that only a relatively small number of samples from an animal infection experiment were available for testing. A larger field study will give more insight into the performance of rapid tests. Furthermore, in this study, the setup of the experimental infection and the timing of swabs arriving in the laboratory unavoidably gave some information on the status of the swabbed animals to the experimenters. In a field study, however, samples will be tested blindly. In the present study, the DSe and DSp of the rapid tests may be overestimated as they were established with samples from an infection experiment, with the tests being performed under ideal conditions in a laboratory. However, it is likely that the DSe and DSp are also high under field conditions for HPAIV that causes disease and mortality in poultry.

In conclusion, PCR by NLRs remains essential and obligatory for the detection of avian influenza, but additional diagnostics such as rapid tests have potential as supportive diagnostic tools in outbreak situations. The LODs of the Clungene, Biopanda, and Anigen rapid tests are sufficient to detect AIV in birds that have a Ct value common in poultry infected with HPAIV. All three tests can detect the different AIV subtypes that can occur in birds, showing that future outbreaks with other variants in the field are likely to be detectable. Furthermore, the DSe is high for samples from experimentally infected birds, and the predicted DSe of the Clungene rapid test for field samples (based on Ct values) is also high. Given the imperfect DSe of this test, its application is only appropriate at the flock level. Assessing the value of applying rapid tests in the field is required to establish if on-site testing can have a place in the fight against avian influenza.

## SUPPLEMENTAL MATERIAL

Supplemental tables and figure spectrum.00654-25-s0001.docx Tables describing an inventory of rapid tests for avian influenza, the analytical sensitivity of a selection of tests, the predicted diagnostic sensitivity for field samples of one test, and a figure summarizing Ct values of poultry field samples.

ASM does not own the copyrights to Supplemental Material that may be linked to, or accessed through, an article. The authors have granted ASM a non-exclusive, world-wide license to publish the Supplemental Material files. Please contact the corresponding author directly for reuse.
