# Supplementary material for: Evaluation of commercial rapid tests for fast and on-site detection of high-pathogenicity avian influenza H5 virus in poultry
Source: Microbiol Spectr. 2025 Aug 27;13(10):e00654-25. doi: 10.1128/spectrum.00654-25 (PMC12502734; doi:10.1128/spectrum.00654-25)
Supplement: Supplemental tables and figure — Tables describing an inventory of rapid tests for avian influenza, the analytical sensitivity of a selection of tests, the predicted diagnostic sensitivity for field samples of one test, and a figure summarizing Ct values of poultry field samples. [file spectrum.00654-25-s0001.docx]

**Supplemental files**

**Supplemental Table 1A. Rapid tests for poultry.** A search returned 38 rapid tests from 22 different manufacturers (or resellers). Four tests meet the criteria of being for AIV and originating from Europe. Availability was confirmed by the manufacturers (early 2023). These were the initially preferred rapid tests (highlighted green). The second groups of six tests were for human samples, and are not available anymore or a duplicate from the first group of four tests. The third group of 12 contains tests produced outside Europe.

| Manufacturer / distributor | Origin | Specified target | Remarks | Format (dipstick, cassette) | Target species | Matrix (trachea, cloaca, other) |
| --- | --- | --- | --- | --- | --- | --- |
| Abbexa | UK / USA | AIV | Production in UK | c | avian | t, c |
| Biopanda | UK | AIV | Also sell human Influenza A/B test | c | avian | t, c |
| Megacor | Austria | AIV | sold by Megacor | d | avian | t, c |
| Novidia (Zoetis) | France / USA | AIV | Formerly Synbiotics, production in USA | d | avian | t, c |
| Arbor Vita | USA | A/H7N9 A/H5N1 | Distribution unknown, target NS1 | c | human | ? |
| Certest | Spain | Influenza A | Also sell human Influenza A/B test | c | human | ? |
| CTK Biotech | USA | Influenza A/B | See table human tests | d | human | ? |
| Nano Diagnostics | USA | AIV | Product disappeared from website | c | avian | c, eye |
| Selinion | France | AIV? | Product disappeared from website | - | - | - |
| Woodley | UK | AIV | UK distributor for Synbiotics, see Novidia | d | avian | t, c |
| Anigen / Bionote | South Korea | AIV H5 | Distribution by Gentaur, Belgium | c | avian | t, c |
| Elabscience | China | AIV H5 H7 H9 | Distribution unknown | c | avian | t, c |
| Hangzhou Evegen | China | AIV | Online shop | c | avian | secretions eye, t, c |
| Krishgen | India | AIV H5 H7 | Site unclear about nature of test (ELISA?), distribution unknown | ? | avian | ? |
| Lilif / iNtRON Bio | South Korea | AIV H5 H9 | Distributors in Europe | c | avian | t, c |
| PetX | India | AIV | Only very large batches, distributed by Indiamart | ? | avian | ? |
| PetX | India | AIV | Distributors in UK | ? | avian | secretions, serum, spleen |
| Quicking Biotech | China | AIV H5 H7 | Distribution unknown | c | avian | secretions or serum |
| Ringbio | China | AIV H5 H7 | Online shop | c | avian | throat |
| Shenzhen | China | AIV H5 H7 H9 | Distribution by Gentaur, Belgium | c | avian | t, c |
| Skyer Diagnostics | South Korea | AIV | Distribution unknown | c | avian | faeces |
| Vet Diagnostix | China | AIV | Online shop | c | avian | Secretions, t, c or faeces |

**Supplemental Table 1B**. **Rapid tests for human samples**. Only rapid tests for influenza virus that did not include other viruses (e.g. SARS-CoV-2, RSV) were selected. The 25 tests found were divided into three groups. Of all tests for which descriptions have been found in the literature regarding use for the detection of influenza in poultry, the availability is indicated (*available, #not available, or unclear, or changed test). Nothing was found in the literature about the third group of rapid tests ($) regarding use for detecting influenza virus in poultry. The first group of assays (highlighted green) may be suitable for the intended work.

| Manufacturer | Origin | Rapid test | Website, remarks |
| --- | --- | --- | --- |
| Abbott | USA | * BinaxNOW Influenza A&B | y, waterproof carton test |
| Quidel | USA | * Quickvue Influenza A+B Test | y |
| Thermo Scientific | USA | * Remel Xpect Flu A & B Test | y |
| Becton Dickinson | USA? | # BD Directigen EZ Flu A+B | n, not available? |
| Coris Bioconcept | Belgium | # Influ-A | n, not available |
| Fujirebio | Japan | # Espline Influenza A&B-N | n, unfindable |
| Genzyme Diagnostic (Sekisui) | Japan | # Rapid Test Flu II | n, unfindable |
| Sysmex Corporation | Japan | # Poctem S Influenza | n, unfindable |
| Accubiotech | China | Accu-Tell Influenza A+B Cassette | y, $ |
| Acon | China | Flowflex Influenza a-b Rapid Test | y, $ |
| Attogene | USA | Influenza A+B Antigen Rapid Test Kit | y, $ |
| Babio | China | Influenza A/B Antigen Detection Kit | y, $ |
| Becton Dickinson | USA | Veritor Plus System for Flu A + B | y, $ nature of test unclear |
| Biopanda | VUK | Influenza A+B Rapid Test | y, $ |
| Biozek | Netherlands | Influenza Rapid Test, both A+B and A | y, $: dipstick and cassette |
| CerTest | Spain | CerTest Influenza A+B one step card test | y, $ |
| Cleartest | USA or Germany | Cleartest influenza A+B | y, $ |
| Clonatest / Linear Chemicals | Spain | Influenza A+B Cassette | y, $ |
| Coris Bioconcept | Belgium | Influ A+B K-SeT | y, $ |
| CTK Biotech.com | USA | Influenza A/B Rapid Test | y, $ |
| Dialab | Austria | Diaquick Rapid Test Influenza Ag Dipstick | y, $ |
| Intec | China | Rapid Flu Test | y, $, A, B, A/B available |
| JusChek | Malaysia | Flu A+B | y, $ |
| Medical disposable | USA | Influenza A+B Test Kit | y, $ |
| Novacyt | UK | PathFlow Influenza A&B | y, $ |

**Supplemental Table 1C. Published use of rapid tests for influenza in poultry**. A chronological overview of studies in which rapid tests (older tests include enzyme immunoassays) have been used for detection of influenza virus in poultry, both experimental infections and field studies.

| Reference | Manufacturer / distributor / name of test in paper | Current name of test and availability / remarks | Human / avian test | Samples tested |
| --- | --- | --- | --- | --- |
| Cattoli et al., 2004 | Becton Dickinson, Directigen | Obsolete, enzymatic assay | h | Pooled trachea swabs of experimentally infected turkeys (AIV H7N3) and of field samples (various poultry species). |
| Woolcock et al., 2005 | QuickVue Influenza test kit | QuickVue Influenza A+B, Quidel | h | AIV H6N2 in allantoic fluid and in oropharyngeal and cloacal swabs from experimentally infected chickens. |
|  | NOW FLU A Test (Binax) | BinaxNOW Influenza A&B, Abbott |  |  |
|  | Zstat Flu (ZymeTx, Inc.) | Obsolete, enzymatic assay |  |  |
|  | FLU OIA (ThermoBiostar) | Unfindable / Thermofisher USA? |  |  |
|  | Directigen Flu A (Becton Dickinson) | Obsolete, enzymatic assay |  |  |
| Chan et al., 2007 | QuickVue Influenza A+B | QuickVue Influenza A+B, Quidel | h | Dilution series of H1N1, H3N2 and AIV H5N1. |
|  | Emergo, BinaxNow Influenza A&B | BinaxNOW Influenza A&B, Abbott |  |  |
|  | Becton Dickinson, DirectigenFlu A+B | Obsolete |  |  |
|  | Becton Dickinson, Directigen EZ Flu A+B |  |  |  |
|  | Sysmex, Poctem Influenza A/B | Obsolete |  |  |
|  | Genzyme Diagnostic, Rapid Test FluII | Obsolete |  |  |
| Chua et al., 2007 | Synbiotics, Flu Detect Influenza A | FluDETECT Avian, Zoetis | a | Swabs from AIV H5N1 infected field samples (poultry and wild birds; swabs from a.o. cloaca, combined trachea + cloaca, feces). |
| Chen et al., 2008 | QuickVue Influenza A+B | QuickVue Influenza A+B, Quidel | h | Dilution series of AIV H5N1 isolates. |
| Das et al., 2008 | Synbiotics, Flu Detect Influenza A | FluDETECT Avian, Zoetis | a | Trachea swabs from experimentally infected chickens (AIV H5N1). |
| Loth et al., 2008 | AnigenR Rapid AIV Ag test | Rapid AIV Ag Test Kit, Anigen | a | Field samples, oropharyngeal and cloaca swabs of free-ranging chickens. |
|  | Synbiotics, Flu Detect Antigen Capture test | FluDETECT Avian, Zoetis |  |  |
| Chen et al., 2010 | Quidel, QuickVue Influenza A+B | QuickVue Influenza A+B, Quidel | h | Dilution series of H5N1 isolates, trachea an cloaca samples from chickens (poultry markets and farms). |
| Lu et al., 2010 | Inverness, BinaxNOW Influenza A&B | BinaxNOW Influenza A&B, Abbott | h | AIV H5N1 in field samples. |
| Marché et al., 2010 | Synbiotics Flu Detect | FluDETECT Avian, Zoetis, via Novidia | a | Dilution series of a H5N1 HPAIV isolate, oral and cloaca swabs from experimentally infected chickens (H5N1 HPAIV). |
|  | Anigen AIV ag | Rapid AIV Ag Test Kit, Anigen |  |  |
|  | Anigen H5 AIV ag | H5 specific rapid test |  |  |
|  | Remel X/pect Flu A&B | Via Thermo Scientific | h |  |
|  | Coris Bioconcept Influ-A | Obsolete |  |  |
|  | Megacor AIV ag | FASTest® AIV Ag, Megacor | a |  |
| Meseko et al., 2010 | Anigen Rapid AIV Ag | Rapid AIV Ag Test Kit, Anigen | a | Cloaca swabs of avian carcasses suspected of HPAIV. |
| Soliman et al., 2010 | Quidel QuickVue Influenza | QuickVue Influenza A+B, Quidel | h | Trachea and cloaca swabs of poultry, mainly chickens, upon suspicion of infection. |
|  | Anigen AIV ag | Anigen Rapid AIV Ag Test Kit | a |  |
| Slomka et al., 2011 | Quidel QuickVue Influenza | QuickVue Influenza A+B, Quidel | h | Trachea and cloaca swabs of chickens and ducks from flocks confirmed to be infected with AIV. |
|  | Anigen AIV ag | Rapid AIV Ag Test Kit, Anigen | a |  |
| Robyn et al., 2012 | Anigen AIV Ag Test | Rapid AIV Ag Test Kit, Anigen | a | Oropharyngeal swabs from chickens during AI outbreak investigations. |
| Loth et al., 2015 | Anigen Rapid AIV Ag Test | Rapid AIV Ag Test Kit, Anigen | a | Oropharyngeal swabs from ducks acquired on a market, from experimentally infected ducks (H5N1). |
| Amen et al., 2015 | Anigen H5, H7, and H9 subtype-specific test kits | Anigen (Bionote) | a | Trachea and cloaca swabs from H5N2 vaccinated chickens during outbreaks with H5N1 AIV. |
| Ssematimba et al., 2018 | Synbiotics Flu Detect | FluDETECT Avian, Zoetis | a | Oropharyngeal swabs from experimentally infected chickens (H5 and H7 LPAIV). |
| Moriguchi et al., 2021 | Espline Influenza A&B-N, Fujirebio Inc | unfindable | h | Surveillance of wild birds. |
|  | Poctem S Influenza, Sysmex Corporation |  |  |  |
| Denzin et al., 2022 | Megacor FASTest® AIV Ag | FASTest® AIV Ag, Megacor | a | Trachea swabs during outbreak investigations (HPAI H5N8). |

**Supplemental Table 1C (continued).**

| References |
| --- |
| 1. Cattoli G, Drago A, Maniero S, Toffan A, Bertoli E, Fassina S, Terregino C, Robbi C, Vicenzoni G, Capua I (2004) Comparison of three rapid detection systems for type A influenza virus on tracheal swabs of experimentally and naturally infected birds. Avian Pathol 33:432-437 2. Woolcock P R, Cardona C J (2005) Commercial immunoassay kits for the detection of influenza virus type A: evaluation of their use with poultry. Avian Dis 49:477-481 3. Chan KH, Lam SY, Puthavathana P, Nguyen TD, Long HT, Pang CM, Chan KM, Cheung CY, Seto WH, Peiris JS (2007) Comparative analytical sensitivities of six rapid influenza A antigen detection test kits for detection of influenza A subtypes H1N1, H3N2 and H5N1. J Clin Virol 38:169-171 4. Chua T H, Ellis T M, Wong CW, Guan Y, Ge SX, Peng G, Lamichhane C, Maliadis C, Tan S W, Selleck P, Parkinson J (2007) Performance evaluation of five detection tests for avian influenza antigen with various avian samples. Avian Dis 51:96-105 5. Chen Y, Xu F, Fan X, Luo H, Ge S, Zheng Q, Xia N, Chen H, Guan Y, Zhang J (2008) Evaluation of a rapid test for detection of H5N1 avian influenza virus. J Virol Methods 154:213-215 6. Das A, Spackman E, Thomas C, Swayne DE, Suarez D L (2008) Detection of H5N1 high-pathogenicity avian influenza virus in meat and tracheal samples from experimentally infected chickens. Avian Dis 52:40-48 7. Loth L, Prijono WB, Wibawa H, Usman TB (2008) Evaluation of two avian influenza type A rapid antigen tests under Indonesian field conditions. J Vet Diagn Invest 20:642-644 8. Chen Y, Xu F, Gui X, Yang K, Wu X, Zheng Q, Ge S, Yuan Q, Yeo AE, Zhang J, Guan Y, Chen H, Xia N (2010) A rapid test for the detection of influenza A virus including pandemic influenza A/H1N1 2009. J Virol Methods 167:100-102 9. Lu H, Ismail M, Khan O A, Al Hammad Y, Abdel Rhman S, Al-Blowi MH (2010) Epidemic outbreaks, diagnostics, and control measures of the H5N1 highly pathogenic avian influenza in the Kingdom of Saudi Arabia, 2007-08. Avian Dis 54:350-356 10. Marché S, van den Berg T (2010) Evaluation of rapid antigen detection kits for the diagnosis of highly pathogenic avian influenza H5N1 infection. Avian Dis 54:650-654 11. Meseko CA, Oladokun AT, Ekong PS, Fasina FO, Shittu IA, Sulaiman L K, Egbuji AN, Solomon P, Ularamu HG, Joannis TM (2010) Rapid antigen detection in the diagnosis of highly pathogenic avian influenza (H5N1) virus in Nigeria. Diagn Microbiol Infect Dis 68:163-165 12. Soliman M, Selim A, Coward VJ, Hassan MK, Aly M, Banks J, Slomka MJ (2010) Evaluation of two commercial lateral flow devices (LFDs) used for flockside testing of H5N1 highly-pathogenic avian influenza infections in backyard gallinaceous poultry in Egypt. J Mol Genet Med 4:247-251 13. Slomka MJ, To T L, Tong H, Coward VJ, Mawhinney IC, Banks J, Brown IH (2011) Evaluation of lateral flow devices for identification of infected poultry by testing swab and feather specimens during H5N1 highly pathogenic avian influenza outbreaks in Vietnam. Influenza Other Respir Viruses 6:318-327 14. Robyn M, Priyono W B, Kim LM, Brum E (2012) Diagnostic sensitivity and specificity of a participatory disease surveillance method for highly pathogenic avian influenza in household chicken flocks in Indonesia. Avian Dis 56:377-380 15. Loth L, Long P, Tung N, Dang N, Newman S (2015) Test Characteristics of the Anigen® Rapid AIV Ag Test (Avian Influenza Type A Rapid Antigen Test) in Ducks in Vietnam. Virol Mycol 4:2161-0517.1000140 16. Amen O, Vemula SV, Zhao J, Ibrahim R, Hussein A, Hewlett I K, Moussa S, Mittal SK (2015) Identification and characterization of a highly pathogenic H5N1 avian influenza A virus during an outbreak in vaccinated chickens in Egypt. Virus Res 210:337-343 17. Ssematimba A, Malladi S, Bonney PJ, Flores-Figueroa C, Muñoz-Aguayo J, Halvorson DA, Cardona C J (2018) Quantifying the effect of swab pool size on the detection of influenza A viruses in broiler chickens and its implications for surveillance. BMC Vet Res 14:265 18. Moriguchi S, Hosoda R, Ushine N, Kato T, Hayama SI (2021) Surveillance system for avian influenza in wild birds and implications of its improvement with insights into the highly pathogenic avian influenza outbreaks in Japan. Prev Vet Med 187:105234 19. Denzin N, Bölling M, Pohlmann A, King J, Globig A, Conraths FJ (2022) Investigation into a Superspreading Event of the German 2020-2021 Avian Influenza Epidemic. Pathogens 11:309 |

**Supplemental Table 1D. Preselection of rapid tests.** The table provides information on a pre-selection of nine rapid tests, divided into three categories (blue, avian; orange, for humans but reported to be used for poultry; yellow, for humans but reported to be used for wild birds). Properties that makes tests less suitable for on-site use during AIV outbreak investigations are indicated in red.

| \|  \| Abbexa \| Biopanda \| Megacor \| Zoetis (Novidia) \| Anigen \| \| --- \| --- \| --- \| --- \| --- \| --- \| \| Price (€, indicative) \| <10 \| <10 \| <10 \| <10 \| <10 \| \| Target species \| poultry \| chicken, duck \| poultry, wild birds \| chicken, turkey \| chicken, duck \| \| Matrix \| trachea / cloaca \| \| \| \| \| \| Format \| cassette \| \| dipstick \| \| cassette \| \| Protocol \| standard: swab in assay buffer (a vial assay for each test), mix, 2-3 droplets on test \| standard: swab in assay buffer (a vial for each test), mix, 3 droplets on test \| swab in assay buffer (a vial for each test), mix, transfer 10 droplets to new tube, insert dipstick \| insert swab in 8 droplets assay buffer (one stock for all tests), mix, insert dipstick \| standard: swab in assay buffer (a vial for each test), mix, 4-5 droplets on test \| \| Reading (min) \| 10-15, <30 \| 15, <20 \| 20, <30 \| 15, <20 \| 20, <30 \| \| DSe \| >98% \| 90,00% \| 100% \| 100% \| 100% \| \| DSp \| >98% \| 98,13% \| 100% \| 100% \| 100% \| \| LOD \| n.d. \| 3.0x10^4^ TCID50 / Test (inactivated H5N1) \| 2^12^-2^15^ HAU of H1-H13 (hemagg. titer 2^8^-2^10^) \| 10^3^-10^5^ EID50/mL in chicken samples \| LOD EID50/ml 10exp4.5-3.5 \| \| Reactivity (only for avian tests) \| unknown \| H5N1, H9N2, H7N9, H7N8, H10N4, H1N1, H3N2 \| AIV subtypes H1–H15 \| all 16 subtypes of AIV Type A \| all AIV type A \| \| License/validation for poultry \|  \|  \| FLI registration (2006) \| USDA-licensed (2006) \| Validated by VLA, FLI, CSIRO Austr. An. Health \| \| Disdvantages \| usage not published \| usage not published \| two sequential tubes \| one stock assay buffer for all tests \| not EU/USA) \| \| incomplete specifications \|  \| hardly reported in literature \|  \| availability currently unclear (january 2025) \| \| Advantages \| designed for poultry, produced in EU/USA \| \| \| \| \| \|  \|  \|  \| appear in multiple publications \| \| \|  \|  \| used in 42 countries (according to manufacturer) \| performance extensively described by manufacturer \| in veterinary literature the most frequent used test \| \|  \|  \|  \|  \|  \|  \| \|  \| Abbott \| Remel (ThermoFisher) \| Quidel \| Clungene \| \| Price (€, indicative) \| >€20 \| >€20 \| <10 \| < €3 \| \| Target species \| human \| \| \| \| \| Matrix \| nose/throat \| \| \| \| \| Format \| cassette-like \| two-lane cassette \| dipstick \| two-lane cassette \| \| Protocol \| swab, assay buffer (vial per test), add 100 ul to test, handle/fold the test \| swab, 25 droplets of assay buffer (one stock for all tests), mix, 100 ul per lane \| swab in assay buffer (a vial for each test), mix, insert dipstick \| standard: swab in assay buffer (a vial for each test), mix, 3 droplets on test \| \| Reading (min) \| 15 \| 15 \| 10 \| 15, <20 \| \| DSe \| 70% \| 88,90% \| 94% \| 89% \| \| DSp \| 90% \| 100% \| 90% \| 99,80% \| \| LOD \| 10^2^ - 10^6^ CEID50/mL \| 1.63 x 10^3^ - 2.0 x 10^5^ TCID50/ml \| H1-H3 pfu/mL: 6.6x10^-1^ - 1.6x10^7^; H7N9: 7.9x10^6^ EID50/mL \| 1,0×10^3^ - 1,0×10^4^ TCID50/mL \| \| Disdvantages \| expensive \| expensive \|  \| use not published \| \| extra handling required \| one stock assay buffer for all tests \|  \| \| for human samples \| for human samples \| for human samples \| for human samples \| \| Advantages \|  \| in literature as sensitive as the most sensitive avian test \|  \| cheap \| \| appear in multiple publications \| \| \|  \| |  |  |  |  |  |
| --- | --- | --- | --- | --- | --- | --- | --- | --- | --- | --- | --- | --- | --- | --- | --- | --- | --- | --- | --- | --- | --- | --- | --- | --- | --- | --- | --- | --- | --- | --- | --- | --- | --- | --- | --- | --- | --- | --- | --- | --- | --- | --- | --- | --- | --- | --- | --- | --- | --- | --- | --- | --- | --- | --- | --- | --- | --- | --- | --- | --- | --- | --- | --- | --- | --- | --- | --- | --- | --- | --- | --- | --- | --- | --- | --- | --- | --- | --- | --- | --- | --- | --- | --- | --- | --- | --- | --- | --- | --- | --- | --- | --- | --- | --- | --- | --- | --- | --- | --- | --- | --- | --- | --- | --- | --- | --- | --- | --- | --- | --- | --- | --- | --- | --- | --- | --- | --- | --- | --- | --- | --- | --- | --- | --- | --- | --- | --- | --- | --- | --- | --- | --- | --- | --- | --- | --- | --- | --- | --- | --- | --- | --- | --- | --- | --- | --- | --- | --- | --- | --- | --- | --- | --- | --- | --- | --- | --- | --- | --- | --- | --- | --- | --- | --- | --- | --- | --- | --- | --- | --- | --- | --- | --- | --- | --- | --- | --- | --- | --- | --- | --- |

**Supplemental Table 2. Assay characteristics of selected rapid tests as reported by the manufacturers.**

|  |  | Abbexa | Biopanda | Anigen | Quidel | Clungene |
| --- | --- | --- | --- | --- | --- | --- |
| Manual | LOD (number of isolates) |  | 3.0x10^4^ TCID_50_ / test (isolate(s) not mentioned) | 10^4.8^ EID_50_ / ml  (isolate(s) not mentioned) | 1.63x10^3^ - 4.4x10^3^ TCID_50_/ ml (2); 7.90x10^5^ EID_50_ / ml (1); 6.60x10^-1^ - 1.60x10^7^ pfu/ml (32) | 61.0x10^3^ – 1.0x10^4^ TCID_50_/ ml (3) |
|  | Performance (Dse, DSp) |  |  |  | Dse and Dsp are given for human samples – not for birds | Dse and Dsp are given for human samples – not for birds |
|  | Inclusivity |  |  |  | Detection of 24 strains form birds and mammals, including subtypes:  H1N1, H2N2, H3N8, H4N6, H5N1, H5N2, H6N2, H7N7, H8N4, H9N2, H10N7, H11N6, H12N5, H13N6, H14N5, H15N8 | Tested: H1N1, H3N2 |
| Manufacturer information sheet | LOD |  |  | EID_50_ / ml”:  A/ty/It/214845  /02/H7N3 - 10^4.5^  A/ty/It/90302  /05/H5N2 - 10^4.5^  H9N2 H5N8 - 10^4.5^  NWS/33 H1N1 - 10^3.5^ |  |  |
|  | Performance (Dse, DSp) |  | Se 90.00%  Sp 98.13%  Accuracy 97.44% | Sensitivity: 100% by farm (n=19), 77.3% by faeces (n=150)  Specificity: 100% vs. HA PCR (n=1402) |  |  |
|  | Inclusivity |  | Detection of AIV subtypes H5N1, H9N2, H7N9, H7N8, H10N4, H1N1, H3N2 | Detection of all AIV  type A |  |  |
| Pers.comm. |  | LOD not tested  Se >98%  Sp >98% |  |  |  |  |

**Supplemental Table 3. Analytical sensitivity of rapid tests**. Top panel: Five rapid tests were investigated in quadruplicate with half-logarithmic dilution series of AI virus (A/chicken/Netherlands/21038165-006010/2021). The corresponding dilutions in TFB and the EID50/ml titers of the HPAI H5N1 virus used are given. The range of the first series (series 1) differ as they were used to estimate the range of dilutions to be tested (series 2-4). Symbols and abbreviations: + (pink), positive; - (light blue), negative; blank (white), not tested; NC, negative control (TFB). Bottom panel: Results established with virus dilutions were used to calculate limits of detection (LOD) according to Spearman-Kärber. Given are LODs at which 50% of the tests are expected to be positive, expressed in terms of dilution, EID50/ml (with standard deviation), and the Ct value of the M-PCR as derived from a calibration line prepared with known concentrations of the same virus.

|  |  |  | Abbexa | | | | Biopanda | | | | Anigen | | | | Quidel | | | | Clungene | | | |
| --- | --- | --- | --- | --- | --- | --- | --- | --- | --- | --- | --- | --- | --- | --- | --- | --- | --- | --- | --- | --- | --- | --- |
| Dilutions (10^) | Titer EID50/ml (10^) | Ct M-PCR | 1 | 2 | 3 | 4 | 1 | 2 | 3 | 4 | 1 | 2 | 3 | 4 | 1 | 2 | 3 | 4 | 1 | 2 | 3 | 4 |
| stock | 9.94 |  | + | + | + | + |  |  |  |  |  |  |  |  |  |  |  |  |  |  |  |  |
| 0.5 | 9.44 |  | + | + | + | + | + |  |  |  | + |  |  |  | + |  |  |  | + |  |  |  |
| 1.0 | 8.94 |  | + | + | + | - | + |  |  |  | + |  |  |  | + |  |  |  | + |  |  |  |
| 1.5 | 8.44 |  | - | - | - | - | + | + | + | + | + | + | + | + | + | + | + | + | + | + | + | + |
| 2.0 | 7.94 | 18.3 | - | - | - | - | + | + | + | + | + | + | + | + | + | + | + | + | + | + | + | + |
| 2.5 | 7.44 |  | - | - | - | - | + | + | + | + | + | + | + | + | + | + | + | + | + | + | + | + |
| 3.0 | 6.94 | 21.4 | - |  |  |  | + | + | + | + | + | + | + | + | - | - | - | - | + | + | + | + |
| 3.5 | 6.44 |  |  |  |  |  | - | - | - | - | - | - | - | - | - | - | - | - | + | + | - | + |
| 4.0 | 5.94 | 24.7 |  |  |  |  | - | - | - | - | - | - | - | - | - | - | - | - | - | - | - | - |
| 4.5 | 5.44 |  |  |  |  |  | - | - | - | - | - | - | - | - | - | - | - | - | - | - | - | - |
| 5.0 | 4.94 | 28.0 |  |  |  |  | - |  |  |  | - |  |  |  | - |  |  |  | - |  |  |  |
| NC | 0 | no Ct | - | - | - | - | - | - | - | - | - | - | - | - | - | - | - | - | - | - | - | - |
|  |  |  |  |  |  |  |  |  |  |  |  |  |  |  |  |  |  |  |  |  |  |  |
| LOD expressed as dilution (10^) | | | 1.1 | | | | 3.3 | | | | 3.3 | | | | 2.8 | | | | 3.6 | | | |
| LOD expressed as EID_50_/ml (10^) | | | 8.8 | | | | 6.7 | | | | 6.7 | | | | 7.2 | | | | 6.3 | | | |
| Standard deviation | | | 0.1 | | | | 0.0 | | | | 0.0 | | | | 0.0 | | | | 0.1 | | | |
| LOD expressed as Ct value | | | 15.3 | | | | 22.3 | | | | 22.3 | | | | 20.6 | | | | 23.5 | | | |

**Supplemental Table 4. Predicted DSe of the Clungene rapid test specified per reason for submission**. The predicted DSe of the Clungene rapid test for PCR positive field samples (see Supplemental Figure 1), based on the modelled diagnostic sensitivity of the Clungene rapid test as presented in Table 5, is specified per reason of submission (early warning, screening, suspicion).

| Reason of submission | Species | Swabs pooled | Number of pools | Mean Ct (range) | DSe (95% CI) |
| --- | --- | --- | --- | --- | --- |
| Early Warning | chicken | cloaca | 2 | 21.8 (17.4-26.2)  17.4  26.2 | 90.4 (71.6-96.4) |
| Early Warning | chicken | trachea | 2 | 21.6 (21.3-21.9)  21.3  21.9 | 92.0 (76.0-97.6) |
| Screening | chicken | cloaca | 3 | 32.6 (29.8-34.8)  29.8  34.8 | 68.7 (28.7-93.2) |
| Screening | chicken | trachea | 3 | 26.5 (23.6-29.5)  23.6  29.5 | 84.2 (65.6-93.8) |
| Screening | duck | cloaca | 9 | 28.5 (25.2-34.3)  25.2  34.3 | 79.5 (55.8-93.2) |
| Screening | duck | trachea | 29 | 26.8 (21.7-32.9)  21.7  32.9 | 83.2 (62.8-93.9) |
| Suspicion | chicken | cloaca | 264 | 22.5 (15.1-35.1)  15.1  35.1 | 89.2 (68.7-96.5) |
| Suspicion | chicken | trachea | 270 | 22.8 (17.0-35.3)  17.0  35.3 | 90.1 (74.2-96.5) |
| Suspicion | duck | cloaca | 62 | 27.2 (18.3-35.4)  18.3  35.4 | 81.4 (56.8-94.3) |
| Suspicion | duck | trachea | 69 | 22.3 (17.4-32.9)  17.4  32.9 | 90.8 (74.6-96.9) |
| Suspicion | turkey | cloaca | 20 | 24.3 (19.5-29.4)  19.5  29.4 | 87.9 (71.7-95.3) |
| Suspicion | turkey | trachea | 27 | 22.5 (19.2-28.4)  19.2  28.4 | 90.5 (74.0-96.8) |


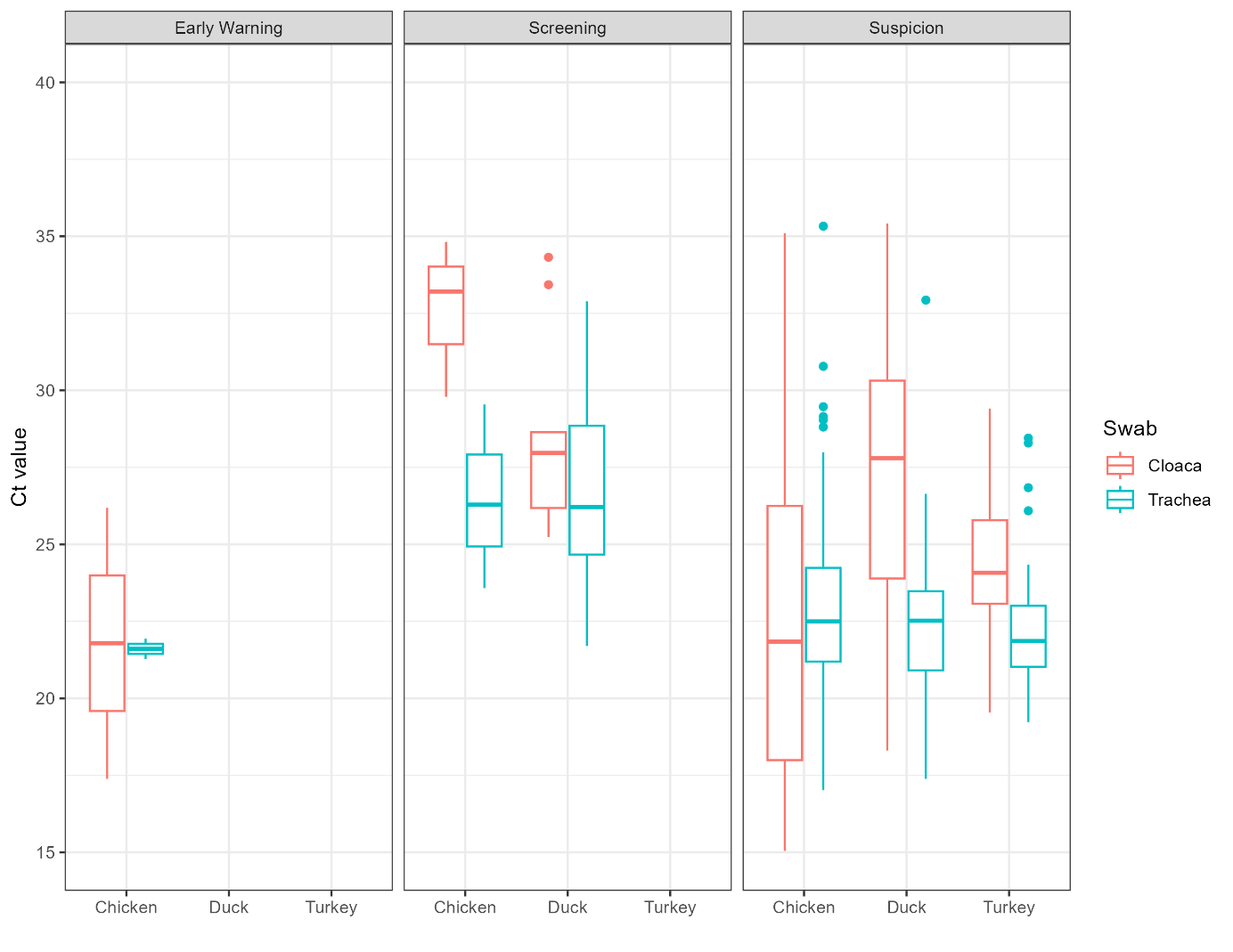


**Supplemental Figure 1. Overview of Ct values of PCR positive field samples.** Results are shown for PCR positive field samples that were tested in the period October 2021 – January 2023. Data are specified per reason of submission (early warning, screening, suspicion) and per bird species (chicken, duck, turkey).
